# Phenotypic analysis and genome sequence of *Rhizopus oryzae* strain Y5, the causal agent of tobacco pole rot

**DOI:** 10.3389/fmicb.2022.1031023

**Published:** 2023-01-04

**Authors:** Zhen Li, Cai-hua Shi, Yang Huang, Han-cheng Wang, Wen-hong Li, Liu-ti Cai

**Affiliations:** ^1^MARA Key Laboratory of Sustainable Crop Production in the Middle Reaches of the Yangtze River (Co-construction by Ministry and Province), College of Agriculture, Yangtze University, Jingzhou, China; ^2^Guizhou Provincial Academician Workstation of Microbiology and Health, Guizhou Academy of Tobacco Science, Guiyang, Guizhou, China; ^3^School of Food Science and Technology & School of Chemical Engineering, Hubei University of Arts and Science, Xiangyang, China; ^4^China Tobacco Sichuan Industrial Corporation Technical Centre, Chengdu, China; ^5^Guizhou Institute of Plant Protection, Guizhou Academy of Agricultural Sciences, Guiyang, Guizhou, China

**Keywords:** *Rhizopus oryzae*, whole genome sequence, tobacco pole rot, biolog phenotype MicroArray, metabolic fingerprint

## Abstract

*Rhizopus oryzae* is a destructive pathogen that frequently causes tobacco pole rot in curing chambers. Phenotypic characterization of the pathogen was conducted to provide basic biological and pathological information using Biolog Phenotype MicroArray (PM). In addition, the Y5 strain of *R. oryzae* was sequenced using Illumina HiSeq and Pacific Biosciences (PacBio) technologies. Using PM plates 1–8, 758 growth conditions were tested. Results indicated that *R. oryzae* could metabolize 54.21% of tested carbon sources, 86.84% of nitrogen sources, 100% of sulfur sources, and 98.31% of phosphorus sources. About 37 carbon compounds, including D-xylose, N-acetyl-D-glucosamine, D-sorbitol, β-methyl-D-glucoside, D-galactose, L-arabinose, and D-cellobiose, significantly supported the growth of the pathogen. PM 3 indicated the active nitrogen sources, including Gly-Asn, Ala-Asp., Ala-Gln, and uric acid. PM 6–8 showed 285 different nitrogen pathways, indicating that different combinations of different amino acids support the growth of the pathogen. Genome sequencing results showed that the *R. oryzae* Y5 strain had raw data assembled into 2,271 Mbp with an N50 value of 10,563 bp. A genome sequence of 50.3 Mb was polished and assembled into 53 contigs with an N50 length of 1,785,794 bp, maximum contig length of 3,223,184 bp, and a sum of contig lengths of 51,182,778 bp. A total of 12,680 protein-coding genes were predicted using the Nonredundant, Gene Ontology, Clusters of Orthologous Groups, Kyoto Encyclopedia of Genes and Genomes, and SWISS-PROT databases. The genome sequence and annotation resources of *R. oryzae* provided a reference for studying its biological characteristics, trait-specific genes, pathogen-host interaction, pathogen evolution, and population genetic diversity. The phenomics and genome of *R. oryzae* will provide insights into microfungal biology, pathogen evolution, and the genetic diversity of epidemics.

## Introduction

Tobacco (*Nicotiana tabacum* L.) is a leafy, annual, solanaceous plant grown commercially for its leaves (Chen et al., 2020). It is one of the most widely grown commercial non-food crops in the world (Liu et al., 2017). Tobacco pole rot has been described as a postharvest pathogen (Chen et al., 2020) as it frequently infects tobacco leaves when that temperature was above 35°C, which occurs in the curing chamber, and had not been shown to infect tobacco leaves in the field. Yearly losses due to tobacco pole rot are immense. In the last 5 years, it became a potentially serious disease of flue-cured tobacco that led to the complete loss of the harvest in southwest China, especially in Guizhou, the second largest tobacco production province. Under high humidity and warm temperatures during curing, leaf rot can damage the whole leaf within the first 2 days in the curing chamber. The first symptoms are white fuzzy spots on the petiole and a watery brown soft rot. Afterward, dark fruiting bodies are formed. These fruiting bodies are filled with spores easily released by the wind (Kortekamp et al., 2003). It frequently occurs during the curing stage, and the disease incidence rate can reach 100% (Surhone et al., 2010).

The fungus of *Rhizopus oryzae* is widely studied, it commonly used for production of some oriental traditional foods, it is mainly recognized as a good producer of lactic acid (Londoño-Hernández et al., 2017). Meanwhile, the pathogen is also the primary cause of mucormycosis, an emerging, life-threatening infection characterized by rapid angioinvasive growth with an overall mortality rate that exceeds 50% (Ma et al., 2009). *R. oryzae*, the causative agent of tobacco pole rot, has also received much attention in recent years. Many studies on the biological characteristics of *R. oryzae* have been conducted. It has a wide range of temperature adaptability, ranging from 25–45°C, with an optimum temperature of 38°C (Chen et al., 2021). Early studies showed that *R. oryzae* was a heat-resistant pathogenic fungus, exhibiting higher growth rates at 25–37°C, and much lower rates were observed at temperatures higher than 40°C (Gayed et al., 1972). Previous studies showed that tobacco pole rot usually occurred during the flue-cured stage, and the termination temperature was about 45°C (Su et al., 2018; Zhang et al., 2018). The pathogen mainly infected petioles and leaves, the pathogenicity varied among different tissues, and the petiole was more conducive to disease (Chen et al., 2021). Carbons such as ribitol, *D*-arabitol, and *ß*-cyclodextrin (Wang et al., 2018a), as well as pH (Chen et al., 2021) affect the growth of *R. oryzae*. However, the metabolic basis for the absence of host specificity by *R. oryzae* is unknown. This includes the absorption and utilization of carbon, nitrogen, phosphorus, and sulfur and whether there is a significant difference in *R. oryzae* infection of tobacco under adverse conditions. Knowing the metabolic phenotype of *R. oryzae* will be valuable to understanding its biochemical properties. It may also help develop potential measures to decrease the overall effect of tobacco pole rot.

Genome sequencing is an important tool for studying the pathogenicity mechanism of plant pathogens. Genomic data are a useful resource to understand the mechanism of plant-pathogen interaction and are used in the phylogenetic analyses of the species (Ailloud et al., 2015). Beyond the isolate of *R. oryzae* from tobacco, many strains of *R. oryzae* were isolated from sweet potato, mulberry, lily (Holmes et al., 2002) and the human body (Nguyen et al., 2020). Information obtained from the National Center for Biotechnology Information (NCBI) could be learned that a total of 43 strains of *R. oryzae* had been sequenced, and there were less differences of genomic data with different *R. oryzae* strains. The genome size ranged from 37.46–55.79 Mb except for strain GL39, with a size of 72.36 Mb. The GC content of most strains was 34%. However, *R. oryzae* has a wide range of hosts. Therefore, more genomic sequences are required to analyze the entire species, especially those isolates from tobacco. To better understand the functions of pathogenicity determinants and the traits of aggressiveness of *R. oryzae* under different ecological environments, the entire genome of the pathogen isolated from tobacco must be sequenced.

Therefore, the objectives of this study were to (i) characterize the metabolic phenotype of *R. oryzae* and (ii) sequence the genome of *R. oryzae*. The genome combined with the metabolic phenotype of the pathogen could provide a reference for the study of fungal biological characteristics, trait-specific genes determination, pathogen-host interaction, pathogen evolution, and population genetic diversity. The data provided by this study will be valuable in expanding our knowledge of the biochemical and metabolic phenomics of *R. oryzae*. It would aid in developing more effective control measures for tobacco pole rot.

## Materials and methods

### Fungal strain and culture conditions

**Figure 1:**
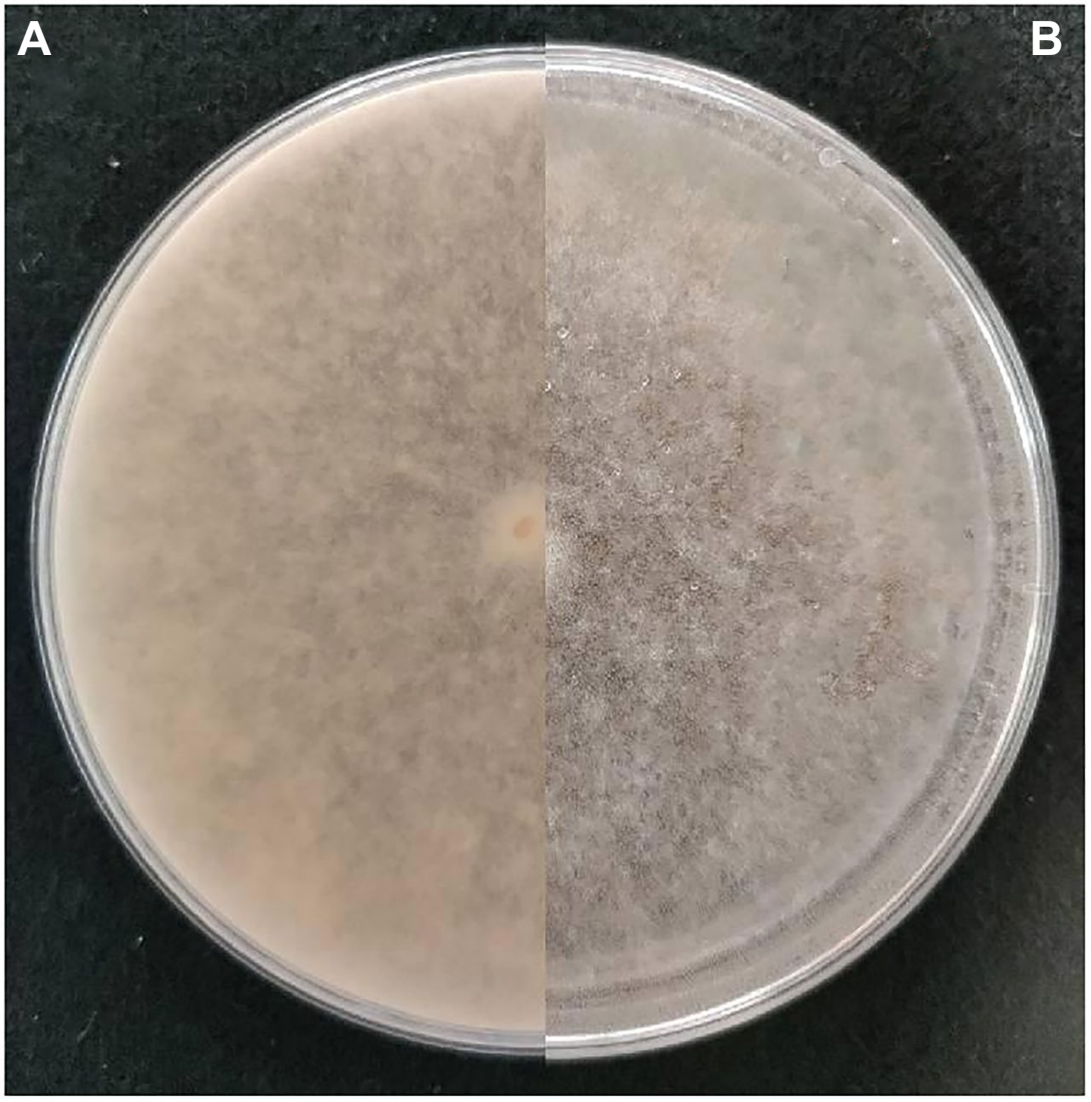
Morphological characteristics of colony of *Rhizopus oryzae* Y5. **(A,B)**. Colony on PDA after 5 days’ incubation at 25°C in the dark (front and reverse).

One isolate of *R. oryzae* strain Y5 (Wang et al., 2016) (GenBank Accession Nos. KT390187) from tobacco was chosen randomly among the isolates of the pathogen (Chen et al., 2020) for analysis from the laboratory of Guizhou Academy of Tobacco Science. It was also conserved at the China Typical Microorganism Conservation Center with conservation No. CCTCC M2015720. The isolate was maintained on potato dextrose agar (PDA), in a controlled climate cabinet at 25°C in darkness. After 5 days of incubation on PDA, conidia were produced (Figure 1), based on the observed colony attributes, the presence of pale brown sporangiospores (5 to 8 μm in diameter) with bluish stripes (Watanabe, 2002). Sterile cotton swabs were moistened with sterile FF Inoculating Fluid (FF-IF), spores were collected by rotating the cotton swabs on the surface of the colony, and then the swabs were mixed into 12 ml FF-IF inoculation solution. The suspension was filtered through a double-layer of sterile cheesecloth (Grade # 40: 24 × 20 threads per inch) to remove mycelial fragments, and the resulting conidia suspension was diluted to a final concentration of 1 × 10^5^ spores mL^−1^.

**Table 1:** Substrates in PM 1–2 carbon source Micro plates significantly supported the growth of *Rhizopus oryzae.*

**Well**	**Substrate**	**Well**	**Substrate**	**Well**	**Substrate**
**PM1**					
A02	*L*-Arabinose	C07	*D*-Fructose	H06	*L*-Lyxose
A03	*N-Acetyl-D-Glucosamine*	C09	*α*-D-Glucose	H09	*L*-Galactonic acid-γ-Lactone
A06	*D*-Galactose	C10	Maltose	H10	*D*-Galacturonic acid
A08	*L*-Proline	D08	*α-Methyl-D-Galactoside*		
A10	*D*-Trehalose	E08	*β*-Methyl-*D*-Glucoside		
A11	*D*-Mannose	E09	Adonitol		
B02	*D*-Sorbitol	E10	Maltotriose		
B08	*D*-Xylose	F11	*D*-Cellobiose		
C04	*D*-Ribose	G05	*L*-Alanyl glycine		
**PM2**					
A05	*γ*-Cyclodextrin	B04	Amygdalin	D08	Xylitol
A06	Dextrin	B05	*D*-Arabinose	G04	*L*-Arginine
A07	Gelatin	B06	*D*-Arabitol	H01	*L*-Ornithine
A08	Glycogen	B08	Arbutin	H09	Dihydroxy acetone
A10	Laminarin	C01	Gentiobiose		
A12	Pectin	D02	Salicin		

### Phenotypic characterization

The metabolic capacity of *R. oryzae* was tested using the Phenotype MicroArray (PM) system (Biolog, Hayward, CA, United States) to determine its phenotype (Bochner et al., 2001; Zhou et al., 2003; Von Eiff et al., 2006). The PM system involved 758 different growth conditions, including 190 diverse carbon sources (PM 1–2), 95 nitrogen sources (PM 3), 285 nitrogen pathways (PM 6–8), 59 phosphorus sources (PM 4), 35 sulfur sources (PM 4) and 94 biosynthetic pathways (PM 5). All materials, media, and reagents for the PM system were purchased from Biolog corporation. In total, 8 PM plates were used in this study. Carbon, nitrogen, phosphorus, sulfur, and biosynthetic pathways were tested for catabolic pathways in Plates 1–8. *R. oryzae* conidia suspension was prepared as detailed above and suspended in a suitable medium containing sterile FF-IF. The turbidity of conidial suspension was tested and was modified to a density of 62% T (transmittance). FF-IF was used for PM plates 1 and 2, FF-IF plus 100 mM D-glucose, 5 mM potassium phosphate (pH 6.0), and 2 mM sodium sulfate was used for PM plates 3 and 5–8. FF-IF plus 100 mM D-glucose was used for PM plate 4 (Wang M. S. et al., 2015). Plates containing 100 μl of the specified mixture were incubated in an OmniLog at 28°C for 1 week, and readings were taken every 15 min. Incubation and recording of phenotypic data were performed in the OmniLog station by capturing digital images of microarrays and storing turbidity values. Kinetic and Parametric software (Biolog, Hayward, CA, United States) was used to analyze the data. The phenotype was estimated according to the area of each well under the staining formation kinetics curve. The experiment was repeated twice.

### Phylogenetic analyses of the sequences of genomic strains of *R. oryzae*

Reference sequences (Supplementary Table S1) from Nguyen et al. (2020) were downloaded from GenBank, the evolutionary history was inferred using the Neighbor-Joining method (Saitou and Nei, 1987), evolutionary analyses were conducted in MEGA7 (Kumar et al., 2016).

### Whole genome sequencing of *R. oryzae* strain of Y5

#### Genome sequencing

##### Extraction of genome DNA

Genomic DNA was extracted with the SDS method (Lim et al., 2016). The harvested DNA was detected by the agarose gel electrophoresis and quantified by Qubit^®^ 2.0 Fluorometer (Thermo Scientific).

#### Library construction

##### Illumina NovaSeq platform

A total amount of 1 μg DNA per sample was used as input material for the DNA sample preparations. Sequencing libraries were generated using NEBNext^®^ Ultra^™^ DNA Library Prep Kit for Illumina (NEB, United States) following manufacturer’s recommendations and index codes were added to attribute sequences to each sample. Briefly, the DNA sample was fragmented by sonication to a size of 350 bp, then DNA fragments were end-polished, A-tailed, and ligated with the full-length adaptor for Illumina sequencing with further PCR amplification. At last, PCR products were purified (AMPure XP system) and libraries were analyzed for size distribution by Agilent2100 Bioanalyzer and quantified using real-time PCR.

##### PacBio sequel platform

Libraries for single-molecule real-time (SMRT) sequencing was constructed with an insert size of 20 kb using the SMRT bell TM Template kit, version 1.0. Briefly, the process was that fragment and concentrate DNA, repair DNA damage and ends, prepare blunt ligation reaction, purify SMRTbell Templates with 0.45X AMPure PB Beads, size-selection using the BluePippin System, repair DNA damage after size-selection. At last, the library quality was assessed on the Qubit^®^ 2.0 Fluorometer (Thermo Scientific) and detected the insert fragment size by Agilent 2,100 (Agilent Technologies).

#### Sequencing

The whole genome of strain Y5 was sequenced using PacBio Sequel platform and Illumina NovaSeq PE150 at the Beijing Novogene Bioinformatics Technology Co., Ltd. The Illumina reads was only used to polish the assembly generated by PacBio reads and helped to reduce gaps and merge contigs.

#### Genome assembly

**Preliminary assembly with SMRT Link v5.0.1** (Ardui et al., 2018; Reiner et al., 2018).

In order to ensure the accuracy of the subsequent analysis results, the low-quality reads were filtered (less than 500 bp) to obtain clean data. Using the automatic error correction function of SMRT portal, the long reads were selected (more than 6,000 bp) as the seed sequence, and the other shorter reads were aligned to the seed sequence by Blasr, so that the accuracy of the seed sequence could be improved further. After assembling we got an initial result.

##### Correct the results of the preliminary assembly

By the variant Caller module of the SMRT Link software, the arrow algorithm was used to correct and count the variant sites in the preliminary assembly results.

#### Genome component prediction

Genome component prediction included the prediction of the coding gene, repetitive sequences and non-coding RNA. The available steps were proceeded as follows:

For Fungi, by default, the Augustus (Stanke et al., 2008) 2.7 program to retrieve the related coding gene was used. Homology reference gene sequences and transcript sequencing data were provided, a complete annotation pipeline, PASA, as implemented at the Broad Institute, involves the following steps: **(A)** *ab initio* gene finding using a selection of the following software tools: GeneMarkHMM, FGENESH, Augustus, and SNAP, GlimmerHMM. **(B)** protein homology detection and intron resolution using the GeneWise software and the uniref90 non-redundant protein database. **(C)** alignment of known ESTs, full-length cDNAs, and most recently, Trinity RNA-Seq assemblies to the genome. **(D)** PASA alignment assemblies based on overlapping transcript alignments from step (C). **(E)** use of EVidenceModeler (EVM) to compute weighted consensus gene structure annotations based on the above (A, B, C, D). **(F)** use of PASA to update the EVM consensus predictions, adding UTR annotations and models for alternatively spliced isoforms (leveraging D and E).

The interspersed repetitive sequences were predicted using the RepeatMasker (Saha et al., 2008).^1^ The tandem Repeats were analyzed by the TRF (Tandem repeats finder; Benson, 1999).

Transfer RNA (tRNA) genes were predicted by the tRNAscan-SE (Lowe and Eddy, 1997). Ribosome RNA (rRNA) genes were analyzed by the rRNAmmer (Lagesen et al., 2007). sRNA, snRNA and miRNA were predicted by BLAST against the Rfam database (Gardner et al., 2009; Nawrocki and Kolbe, 2009).

#### Gene function prediction

Seven databases were used to predict gene functions. They were respective GO (Gene Ontology; Ashburner et al., 2000), KEGG (Kyoto Encyclopedia of Genes and Genomes; Kanehisa et al., 2004, 2006), KOG (Clusters of Orthologous Groups), NR (Non-Redundant Protein Database databases; Li et al., 2002), TCDB (Transporter Classification Database; Milton et al., 2014), P450 (Crešnar and Petrič, 2011) and Swiss-Prot (Amos and Rolf, 2000). A whole genome Blast search (E-value less than 1e-5, minimal alignment length percentage larger than 40%) was performed against above seven databases. The secretory proteins were predicted by the Signal P database (Petersen et al., 2011). Meanwhile, we analyzed the secondary metabolism gene clusters by the antiSMASH (Medema et al., 2011). For pathogenic fungi, we added the pathogenicity and drug resistance analyses. We used the PHI (Martin et al., 2015; Pathogen Host Interactions), DFVF (database of fungal virulence factors) to perform the above analyses. Carbohydrate-Active enzymes were predicted by the Carbohydrate-Active enZYmes Database (Cantarel et al., 2009).

## Results

### Phenotypic characterization of *R. oryzae* strain Y5

**Figure 2:**
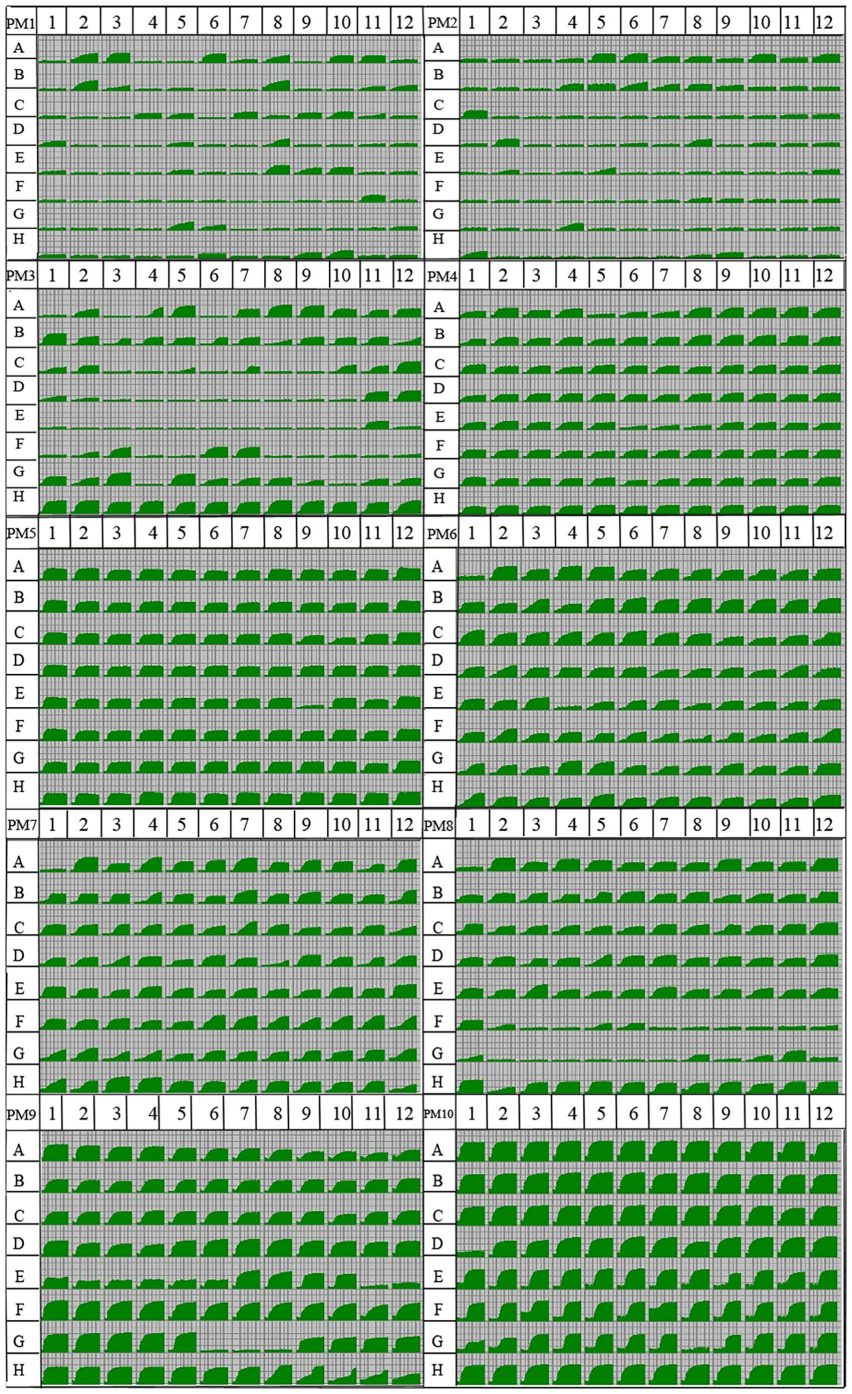
Data for biology phenotype microarray PM 1–8 plates of the pathogen *Rhizopus oryzae.* Utilization of the isolate Y5 of *R. oryzae* from tobacco was indicated by green areas in the growth curve for each substrate, the larger the green area, the higher the utilization. A kinetic response curve which parallels microbial growth can be generated for each well, allowing growth to be compared between samples through multiple parameters such as lag, slope, and area under the curve.

Using the OmniLog PM system, a kinetic response curve which parallels microbial growth can be generated for each well, allowing growth to be compared between samples through multiple parameters such as lag, slope, and area under the curve (Figure 2). The isolate Y5 of *R. oryzae* tested presented a representative phenotypic fingerprint. The fungus was able to metabolize 54.21% of tested carbon sources (51/95 in plate PM1 and 52/95 in plate PM2), 86.84% of nitrogen sources (61/95 in plate PM3, 93/95 in plate PM6, 94/95 in plate PM7, and 82/95 in plate PM8), 100% of sulfur sources (35/35 in plate PM4, wells F02-H12) and 98.31% of phosphorus sources (58/59 in plate PM4, wells A02-E12). The efficient metabolism of carbon sources rate was 19.27%, and the opportune metabolism of carbon sources rate was 17.19%. The efficient metabolism of nitrogen sources rate was 53.91%, and the opportune metabolism of carbon sources rate was 27.34%. The efficient metabolism of sulfur sources rate was 97.14%. The efficient metabolism of phosphorus sources rate was 76.27%, and the opportune metabolism of carbon sources rate was 16.49%. The pathogen presented 94 different biosynthetic pathways (94/94 tested, plate PM5, wells A3-H12).

**Table 2:** Substrates in PM 3, 6–8 nitrogen source Micro plates significantly supported the growth of *Rhizopus oryzae.*

**Well**	**Substrate**	**Well**	**Substrate**	**Well**	**Substrate**
**PM3**					
A05	Urea	E11	*N*-Acetyl-*D*-Glucosamine	H05	Ala-His
A07	*L*-Alanine	F03	Adenosine	H06	Ala-Leu
A08	*L*-Arginine	F06	Guanine	H07	Ala-Thr
A09	*L*-Asparagine	F07	Guanosine	H08	Gly-Asn
A10	*L*-Aspartic acid	G01	Xanthine	H09	Gly-Gln
A12	*L*-Glutamic acid	G03	Uric acid	H10	Gly-Glu
B01	*L*-Glutamine	G05	Allantoin	H11	Gly-Met
B02	Glycine	G08	*γ*-Amino-*N*-Butyric acid	H12	Met-Ala
B09	*L*-Proline	H01	Ala-Asp		
C12	*L*-Ornithine	H02	Ala-Gln		
D11	Putrescine	H03	Ala-Glu		
D12	Agmatine	H04	Ala-Gly		
**PM6**					
A02	Positive control: L-Glutamine	B12	Arg-Lys	E03	Gly-Arg
A03	Ala-Ala	C01	Arg-Met	E07	Gly-Leu
A04	Ala-Arg	C02	Arg-Phe	E12	Gly-Ser
A05	Ala-Asn	C03	Arg-Ser	F01	Gly-Thr
A06	Ala-Glu	C04	Arg-Trp	F02	Gly-Trp
A07	Ala-Gly	C05	Arg-Tyr	F04	Gly-Val
A08	Ala-His	C06	Arg-Val	F12	His-Trp
A09	Ala-Leu	C07	Asn-Glu	G04	Ile-Arg
A10	Ala-Leu	C08	Asn-Val	G05	Ile-Gln
A11	Ala-Phe	C11	Asp-Leu	G09	Ile-Met
A12	Ala-Pro	C12	Asp-Lys	G12	Ile-Ser
B01	Ala-Ser	D01	Asp-Phe	H01	lle-Trp
B02	Ala-Thr	D02	Asp-Trp	H02	Ile-Tyr
B03	Ala-Trp	D03	Asp-Val	H04	Leu-Ala
B05	Arg-Ala	D04	Cys-Gly	H05	Leu-Arg
B06	Arg-Arg	D05	Gln-Gln	H06	Leu-Asp
B07	Arg-Asp	D06	Gln-Gly	H07	Leu-Glu
B08	Arg-Gln	D09	Glu-Gly	H08	Leu-Gly
B09	Arg-Glu	D11	Glu-Trp	H09	Leu-Ile
B10	Arg-Ile	E01	Glu-Val	H11	Leu-Met
B11	Arg-Leu	E02	Gly-Ala	H12	Leu-Phe
**PM7**					
A02	Positive control:L-Glutamine	E04	Ser-Leu	H03	Val-Arg
A04	Leu-Trp	E07	Ser-Pro	H04	Val-Asn
A05	Leu-Val	E10	Ser-Val	H05	Val-Gly
A06	Lys-Ala	E11	Thr-Ala	H06	Val-Gly
A07	Lys-Arg	E12	Thr-Arg	H07	Val-His
A09	Lys-Ile	F01	Thr-Glu	H08	Val-Ile
A10	Lys-Leu	F02	Thr-Gly	H09	Val-Leu
A12	Lys-Phe	F03	Thr-Leu	H10	Val-Tyr
B07	Met-Arg	F06	Trp-Ala	H11	Val-Val
B09	Met-Gln	F07	Trp-Arg		
B12	Met-His	F08	Trp-Asp		
C01	Met-Ile	F10	Trp-Gly		
C02	Met-Leu	F11	Trp-Leu		
C08	Met-Pro	G02	Trp-Ser		
C11	Phe-Ile	G06	Tyr-Gln		
D04	Pro-Ala	G10	Tyr-Leu		
D06	Pro-Gln	G11	Tyr-Lys		
D09	Pro-Leu	G12	Tyr-Phe		
D12	Pro-Tyr	H01	Tyr-Trp		
E01	Ser-Ala	H02	Tyr-Tyr		
**PM8**					
A02	Positive Control:*L*-Glutamine	C01	Lys-Gly	E04	Tyr-lle
A03	Ala-Asp	C03	Met-Phr	E06	Val-Ala
A04	Ala-Gln	C04	Met-Tyr	E07	Val-Gln
A05	Ala-lle	C05	Phe-Asp	E08	Val-Glu
A06	Ala-Met	C07	Gln-Glu	E09	Val-Lys
A07	Ala-Val	C08	Phe-Met	E10	Val-Met
A08	Asp-Ala	C10	Phe-Val	E11	Val-Phe
A09	Asp-Gln	C11	Pro-Arg	E12	Val-Pro
A10	Ala-Gly	C12	Pro-Asn	F01	Val-Ser
A11	Glu-Ala	D01	Pro-Glu	G11	Ala-Ala-Ala
A12	Gly-Asn	D02	Pro-lle	H01	Gly-Gly-Ala
B01	Gly-Asp	D04	Pro-Ser	H03	Gly-Gly-Gly
B02	Gly-lle	D06	Pro-Val	H04	Gly-Gly-IIe
B03	His-Ala	D07	Ser-Asn	H05	Gly-Gly-Leu
B05	His-His	D08	Ser-Asp	H06	Gly-Gly-Phe
B06	lle-Asn	D09	Ser-Gln	H07	Val-Tyr-Val
B07	Ile-Leu	D10	Ser-Glu	H08	Gly-Phe-Phe
B08	Leu-Asn	D11	Thr-Asp	H09	Leu-Gly-Gly
B10	Leu-Pro	D12	Thr-Gln	H10	Leu-Leu-Leu
B11	Leu-Tyr	E01	Thr-Phe	H11	Phe-Gly-Gly
B12	Lys-Asp	E03	Trp-Val	H12	Tyr-Gly-Gly

### Carbon source utilization characteristics of strain Y5

Based on data from PM1 and PM2 (carbon sources), the isolate of *R. oryzae* from tobacco could use 103 different carbon sources and about 37 compounds (Table 1), including *D*-xylose, *N*-acetyl-*D*-glucosamine, *D*-sorbitol, *β*-methyl-*D*-glucoside, *D*-galactose, *L*-arabinose, *D*-cellobiose, *D*-Mannose, *D*-Galacturonic acid, maltotriose, *D*-trehalose, *L*-alanyl glycine, *D*-fructose, maltose, *α*-*D*-glucose, *L*-proline, *D*-ribose, adonitol, *L*-lyxose, *L*-galactonic acid-*γ*-lactone, *α*-methyl-*D*-galactoside, *γ*-cyclodextrin, pectin, dextrin, laminarin, gentiobiose, salicin, *D*-arabinose, *D*-arabitol, dihydroxy acetone, arbutin, glycogen, *L*-ornithine, xylitol, amygdalin, gelatin, and *L*-arginine significantly supported the growth of the pathogen. In comparison, around 87 compounds significantly inhibited the growth of the pathogen. Therefore, the utilization rate of carbon was lower than that of other sources.

### Nitrogen sources utilization characteristics of strain Y5

Based on data from the PM3 plate, the isolate was tested for its ability to grow on 95 different nitrogen sources (amino acids). Sixty one compounds supported the growth of the pathogen, typical compounds included Gly-Asn, Ala-Asp., Ala-Gln, uric acid, Ala-Gly, Gly-Gln, Met-Ala, Gly-Glu, *L*-arginine, *L*-glutamine, *L*-ornithine, Ala-Glu, *L*-asparagine, Ala-Thr, Ala-Leu, allantoin, Gly-Met, agmatine, urea, guanine, Ala-His, guanosine, putrescine, xanthine, *L*-glutamic acid, *L*-aspartic acid, adenosine, *L*-proline, *L*-alanine, *N*-acetyl-*D*-glucosamine, glycine, and *γ*-amino-*N*-butyric acid. In comparison, 34 out of 95 nitrogen sources supported growth in the negative control, indicating that *R. oryzae* cannot metabolize these compounds. Based on PM6 to PM8 (nitrogen pathway) data, *R. oryzae* showed 285 different nitrogen pathways, indicating that different combinations of different amino acids support the growth of the pathogen. The result showed that 260 nitrogen pathways supported the growth of the pathogen, and more than 175 efficient nitrogen pathways, including *L*-glutamine, Arg-Arg, Ala-Arg, Arg-Lys, Ala-Asn, Arg-Met, Arg-Gln, Arg-Ala, Arg-Ile, Arg-Val, Arg-Asp., Ile-Arg, Arg-Glu, Leu-Phe, Arg-Leu, Arg-Ser, Ala-Pro, Arg-Trp, Leu-Arg, Ile-Gln, Gly-Arg, Asn-Glu, Asn-Val, Arg-Phe, Arg-Tyr, Ala-Gly, Ile-Ser, Ala-Trp, Asp-Lys, Ala-Ala, and lle-Trp. In comparison, around 25 compounds significantly inhibited the growth of the pathogen (Table 2). Therefore, the utilization rate of nitrogen sources was higher than that of carbon sources.

### Phosphorus and sulfur sources utilization characteristics of strain Y5

The pathogen presented unapparent growth in the negative control without any phosphorus source (plate PM4, Well A01). Meanwhile, the pathogen could assimilate all S-containing compounds tested (35/35 tested, plate PM4, Wells F1-H12) (Table 3). Typical compounds included tetramethylene sulfone, butane sulfonic acid, *L*-methionine sulfone, hypotaurine, methane sulfonic acid, thiourea, *L*-cysteine sulfinic acid, *D*, *L*-lipoamide, *L*-cysteic acid, *ρ*-amino benzene sulfonic acid, 1-thio-*β*-*D*-glucose, glycyl-*L*-methionine, taurine, *L*-cysteinylglycine, 2-hydroxyethane sulfonic acid, cysteamine, thiosulfate, and cystathionine. The pathogen presented 94 different biosynthetic pathways (94/94 tested, plate PM5, wells A3-H12). Typical biosynthetic pathways included Tween 80, caprylic acid, myo-inositol, Tween 20, 2′-deoxy inosine, *D, L*-carnitine, 2′-deoxy adenosine, 2′-deoxy uridine, *D*, *L*-*α*-lipoic acid (oxidized form), Tween 60, thymidine, butyric acid, and *L*-glutamine (Table 4).

### Phylogenetic analyses

The optimal tree with the sum of branch length = 31.57 was shown. The percentage of replicate trees in which the associated taxa clustered together in the bootstrap test (1,000 replicates) were shown next to the branches (Felsenstein, 1985). The tree was drawn to scale, with branch lengths in the same units as those of the evolutionary distances used to infer the phylogenetic tree. The evolutionary distances were computed using the Maximum Composite Likelihood method (Tamura et al., 2004) and were in the units of the number of base substitutions per site. The analysis involved 37 nucleotide sequences. Codon positions included were 1st + 2nd + 3rd + Noncoding. All positions containing gaps and missing data were eliminated. There was a total of 276 positions in the final dataset (Supplementary Figure S1).

### Whole genome sequencing and statistical analysis

**Figure 3:**
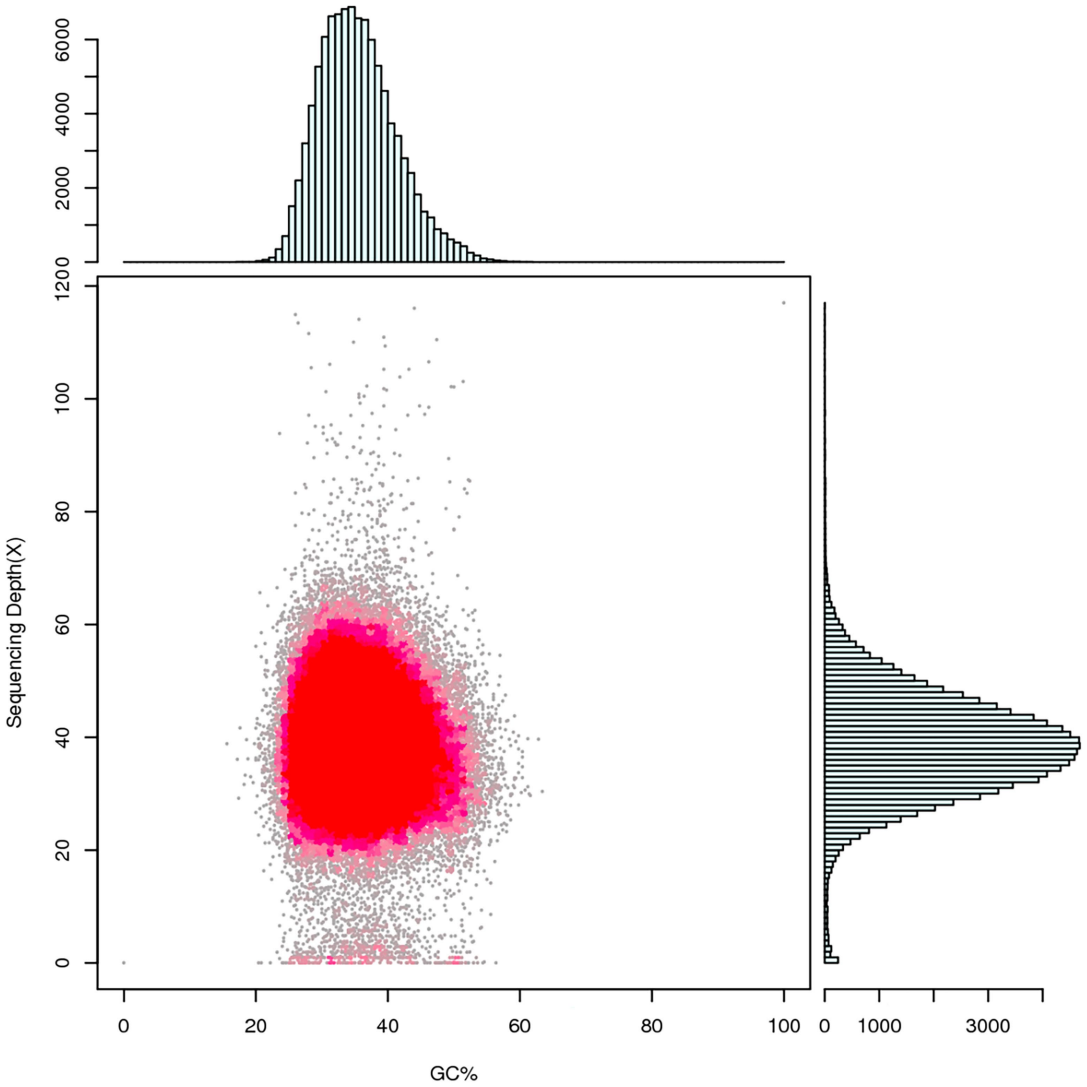
Statistical diagram of correlation analysis between GC content and sequencing depth. Summarize the GC bias and repetitive sequences of the genome by counting the GC content and reads coverage depth of assembled sequences. The horizontal coordinate indicates the GC content, the vertical coordinate indicates the sequencing depth, the right side is the sequencing depth distribution, and the upper side is the GC content distribution.

Whole genome sequencing was performed using single-molecule real-time (SMRT) sequencing on the PacBio RS II (Eid et al., 2009) and HiSeq PE150 platforms. PacBio RS II platform yielded 283,154 reads encompassing 2,129,349,468 bp, with an N50 value of 10,563 bp, using SMRT Link v5.1.0 software^2^ (Ardui et al., 2018; Reiner et al., 2018) for genome assembly to obtain the preliminary result which can reflect the basic condition of the sample genome. A genome sequence of 50.3 Mb was polished and assembled into 53 contigs with an N50 length of 1,785,794 bp, maximum contig length of 3,223,184 bp, and a total contig length of 51,182,778 bp. After the quality control, the number of contigs was 41 with an N50 length of 1,791,927 bp, maximum contig length of 3,235,075 bp, and total length of 50,257,186 bp (~45× genome average coverage; Table 5), and GC content of *R. oryzae* genome was found to be 35.57% which is similar to other forty-three sequenced *R. oryzae* (Figure 3; Supplementary Table S1).

**Table 3:** Substrates in PM 4 phosphorus and sulfur source Micro plates significantly supported the growth of *Rhizopus oryzae.*

**Well**	**Substrate**	**Well**	**Substrate**	**Well**	**Substrate**
**PM4**					
A02	Phosphate	D02	*D*-Mannose-6-Phosphate	G02	*S*-Methyl-*L*-Cysteine
A03	Pyrophosphate	D03	Cysteamine-*S*-Phosphate	G03	Cystathionine
A04	Trimeta-Phosphate	D04	Phospho-*L*-Arginine	G04	Lanthionine
A08	Adenosine-2’-Monophosphate	D05	*O*-Phospho-*D*-Serine	G05	Glutathione
A09	Adenosine-3’-Monophosphate	D06	*O*-Phospho-*L*-Serine	G08	*D*-Methionine
A10	Adenosine-5’-Monophosphate	D07	*O*-Phospho-*L*-Threonine	G09	Glycyl-*L*-Methionine
A11	Adenosine-2′,3’-Cyclic monophosphate	D08	Uridine-2’-Monophosphate	G10	*N*-Acetyl-*D,L*-Methionine
A12	Adenosine-3′,5’-Cyclic monophosphate	D09	Uridine-3’-Monophosphate	G11	*L*-Methionine sulfoxide
B02	Dithiophosphate	D10	Uridine-5’-Monophosphate	G12	*L*-Methionine sulfone
B03	*D,L*-*α*-Glycerol phosphate	D11	Uridine-2′,3’-Cyclic monophosphate	H01	*L*-Djenkolic acid
B04	*β*-Glycerol phosphate	D12	Uridine-3′,5’-Cyclic monophosphate	H02	Thiourea
B05	Carbamyl phosphate	E01	*O*-Phospho-*D*-Tyrosine	H03	1-Thio-*β*-*D*-Glucose
B06	*D*-2-Phospho-Glyceric acid	E02	*O*-Phospho-*L*-Tyrosine	H04	*D,L*-Lipoamide
B07	*D*-3-Phospho-Glyceric acid	E03	Phosphocreatine	H05	Taurocholic acid
B08	Guanosine-2’-Monophosphate	E04	Phosphoryl choline	H06	Taurine
B09	Guanosine-3’-Monophosphate	E05	*O*-Phosphoryl-Ethanolamin	H07	Hypotaurine
B10	Guanosine-5’-Monophosphate	E09	Thymidine-3’-Monophosphate	H08	*p*-Amino benzene sulfonic acid
B11	Guanosine-2′,3’-Cyclic monophosphate	E10	Thymidine-5’-Monophosphate	H09	Butane sulfonic acid
B12	Guanosine-3′,5’-Cyclic monophosphate	F01	Negative control	H10	2-Hydroxyethane sulfonicacid
C01	Phosphoenol pyruvate	F02	Sulfate	H11	Methane sulfonic acid
C02	Phospho-Glycolic acid	F03	Thiosulfate	H12	Tetramethylene sulfone
C03	*D*-Glucose-1-Phosphate	F04	Tetrathionate		
C05	2-Deoxy-*D*-Glucose 6-Phosphate	F06	Dithiophosphate		
C06	*D*-Glucosamine-6-Phosphate	F07	*L*-Cysteine		
C07	6-Phospho-Gluconic acid	F08	*D*-Cysteine		
C08	Cytidine-2’-Monophosphat	F09	*L*-Cysteinyl_x005f glycine		
C09	Cytidine-3’-Monophosphate	F10	*L*-Cysteic acid		
C10	Cytidine-5’-Monophosphate	F11	Cysteamine		
C11	Cytidine-2′,3’-Cyclic monophosphate	F12	*L*-Cysteine sulfinic acid		
C12	Cytidine- 3′,5’-Cyclic monophosphate	G01	*N*-Acetyl-*L*-Cysteine		

**Table 4:** Substrates in PM 5 nutrient supplements Micro plates significantly supported the growth of *Rhizopus oryzae.*

**Well**	**Substrate**	**Well**	**Substrate**	**Well**	**Substrate**
**PM5**					
A01	Negative control	C09	(5) 4-Amino-Imidazole-4 (5)-Carboxamide	F07	Deferoxamine mesylate
A02	Positive control	C11	Inosine	F08	*D*-(+)-Glucose
A03	*L*-Alanine	C12	2’-Deoxy inosine	F09	*N*-Acetyl D-Glucosamine
A04	*L*-Arginine	D01	*L*-Ornithine	F10	Thymine
A05	*L*-Asparagine	D02	*L*-Citrulline	F11	Glutathione (reduced form)
A06	*L*-Aspartic acid	D03	Chorismic acid	F12	Thymidine
A07	*L*-Cysteine	D04	(−)Shikimic acid	G01	Oxaloacetic acid
A08	*L*-Glutamic acid	D05	*L*-Homoserine lactone	G02	*D*-Biotin
A09	Adenosine-3′,5′-cyclic monophosphate	D06	*D*-Alanine	G03	Cyano-Cobalamine
A10	Adenine	D07	*D*-Aspartic acid	G04	P*-Amino-Benzoic acid*
A11	Adenosine	D08	*D*-Alanine	G05	Folic acid
A12	2’-Deoxy adenosine	D09	*D,L-α,ε-Diamino-pimelic acid*	G06	Inosine+Thiamine
B01	*L*-Glutamine	D10	Cytosine	G07	Thiamine
B02	Glycine	D11	Cytidine	G08	Thiamine pyrophosphate
B03	*L*-Histidine	D12	2’-DeoxyCytidine	G09	Riboflavin
B04	*L*-Isoleucine	E01	Putrescine	G10	Pyrrolo-Quinoline Quinone
B05	*L*-Leucine	E02	Spermidine	G11	Menadione
B06	*L*-Lysine	E03	Spermine	G12	Myo-Inositol
B07	*L*-Methionine	E04	Pyridoxine	H01	Butyric acid
B08	*L* Phenylalanine	E05	Pyridoxal	H02	*D, L-α*-Hydroxy-Butyric acid
B09	Guanosine-3′,5′-cyclic monophosphate	E06	Pyridoxamine	H03	*α*-Ketobutyric acid
B10	Guanine	E07	*β*-Alanine	H04	Caprylic acid
B11	Guanosine	E08	*D*-Pantothenic acid	H05	*D, L-α*-Lipoic acid (oxidized form)
B12	2’-Deoxy guanosines	E10	Uracil	H06	*D, L*-Mevalonic acid
C01	*L*-Proline	E11	Uridine	H07	*D, L*-Carnitine
C02	*L*-Serine	E12	2’-Deoxy Uridine	H08	Choline
C03	*L*-Threonine	F01	Quinolinic acid	H09	Tween20
C04	*L*-Tryptophan	F02	Nicotinic acid	H10	Tween40
C05	*L*-Tyrosine	F03	Nicotinamide	H11	Tween60
C06	*L*-Valine	F04	*β*-Nicotinamide adenine dinucleotide	H12	Tween80
C07	*L*-isoleucine + *L*-Valine	F05	*δ*-Amino-Levulinic acid		
C08	trans-4-HydroxyL-Proline	F06	Hematin		

**Table 5:** Genome characteristics and predicted features of the assembled *Rhizopus oryzae* strain Y5.

**Assembly parameters**	**Y5**
Sequencing platform	PacBio and Illumina PE150
Assembly method	SMRT Link v5.0.1
Genome size (Mb)	50.26
Sequencing coverage	45.2 x
Number of contigs	41
Average contig length (bp)	1,225,785
Contig N50 (bp)	1,791,927
Maximum contig length (Mp)	3.24
Number of all Contigs	53
Maximum Contig length(bp)	3,223,184
Contig N50 length (bp)	1,785,794
G + C content (%)	35.57

**Table 6:** Genomic component statistics and transposable element repeat class analysis of *Rhizopus oryzae* strain Y5.

**Gene prediction**				
Gene number	12,680			
Gene total length (bp)	17,290,559			
Gene average length (bp)	1,364			
Gene/genome (%)	34.40			
**Repeat**				
Type	Number	Total Length (bp)	In Genome (%)	Average length (bp)
LTR	9,556	4,996,700	9.9423	527
DNA	4,666	1,615,842	3.2151	350
LINE	2,387	191,492	0.3810	89
SINE	99	6,322	0.0126	64
Unknown	29	2,123	0.0042	73
RC	87	7,851	0.0156	90
Total	16,824	6,789,812	13.5101	410
Type	Number	Repeat Size (bp)	Total Length (bp)	In Genome (%)
**TR**	14,866	1 ~ 1,821	750,593	1.4935
Minisatellite DNA	8,430	10 ~ 60	389,743	0.7755
Microsatellite DNA	961	2 ~ 6	44,535	0.0886
**ncRNA**	Type	Number	Average length (bp)	Total Length (bp)
	tRNA	276	75	20,768
rRNA	5 s (denovo)	3	115	345
	5.8 s (denovo)	0	0	0
	18 s (denovo)	2	1,822	3,645
	28 s (denovo)	3	4,505	13,514
	snRNA	39	118	4,630

### Genome component analysis of coding genes, repeat sequence and ncRNA

The gene prediction was annotated, resulting in 12,680 genes. The total length of genes was 17,290,559 bp, and the average length was 1,364 bp, accounting for 34.4% of the genome. The TEs (transposable elements) represented 13.51% of the genome assembly with a total length of 6,787,689 bp. The total number of TE families analyzed with RepeatMasker in the genome assembly was 16,795 of which 16,708 (99.0%) belonged to the known TEs, including 12,042 retrotransposons (Class I) and 4,666 DNA transposons (Class II; Table 6). Class I retrotransposons could be mainly divided into three groups of TEs, including LINE, LTR, and SINE. Owing to the redundancy of repeats, these duplications were more tolerant of mutations, such as transposon insertions and sequence rearrangements, and might therefore act as a hotspot for genome expansion (Lippman et al., 2004). Tandem Repeat (TR) units with species composition specificity could be used as genetic traits of species for the study of evolutionary relationships. The TR represented 1.49% of the genome assembly with a total length of 750,593 bp. Non-coding RNAs (ncRNA) resembled mRNA in structure and function, they could regulate the transcription and translation of mRNAs in close proximity to them, except that unlike mRNAs that could be translated into proteins. The total number of ncRNA was 323, with a total length of 42,902 bp.; this suggested that ncRNA formed only a small proportion of the overall genome size (Table 6).

### Genome function analysis of pathogenicity related genes

**Figure 4:**
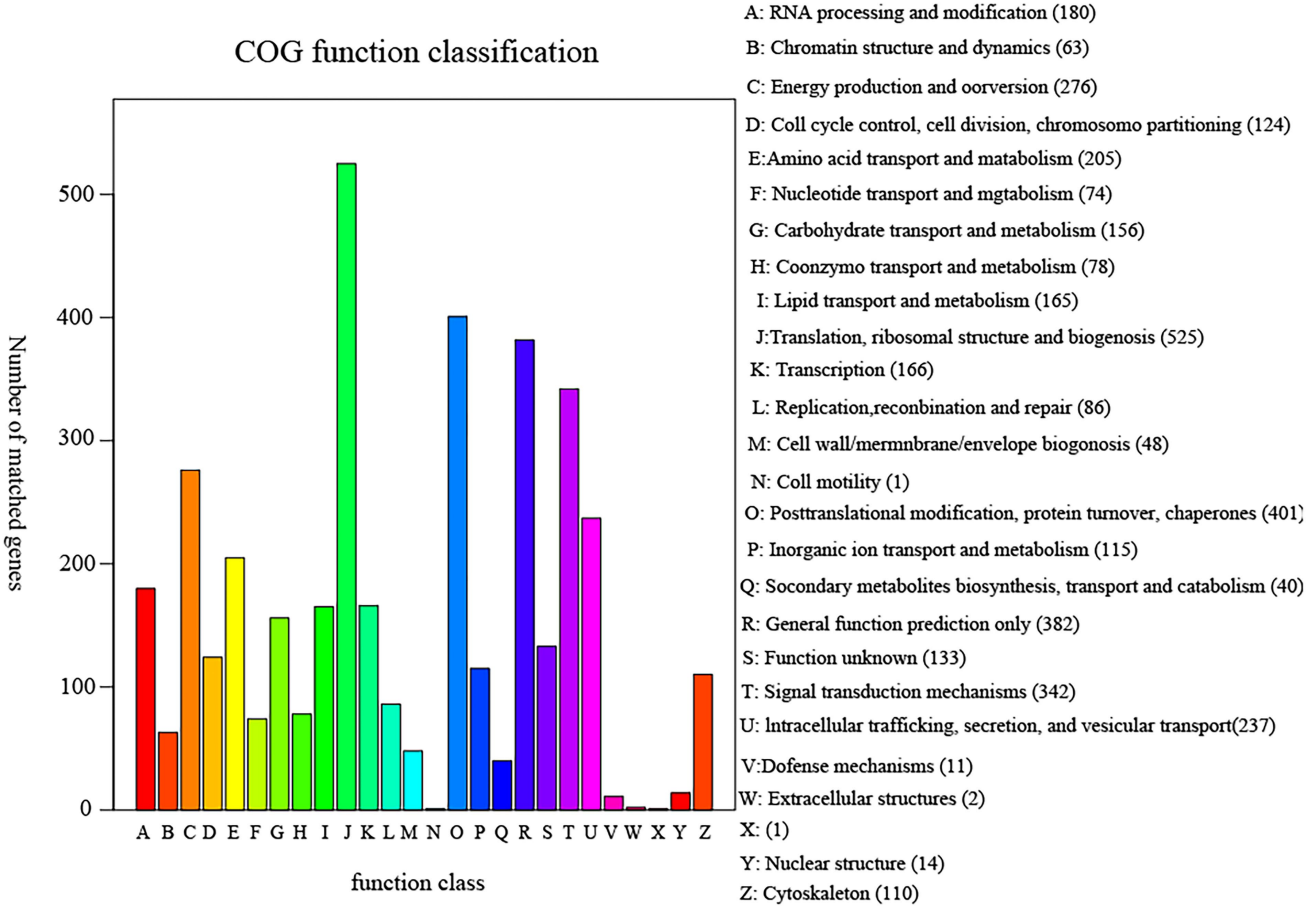
COG functional classification diagram of sample Y5 gene function annotation. COG (Cluster of Orthologous Groups of proteins) is constructed based on the classification of phylogenetic relationships of proteins encoded in complete genomes of bacteria, algae and eukaryotes. By comparison, a protein sequence can be annotated to a particular COG, and each cluster of COGs consists of direct homologous sequences, allowing the function of that sequence to be inferred. The abscissa represents the type of COG function and the ordinate represents the number of genes on the annotation.

**Figure 5:**
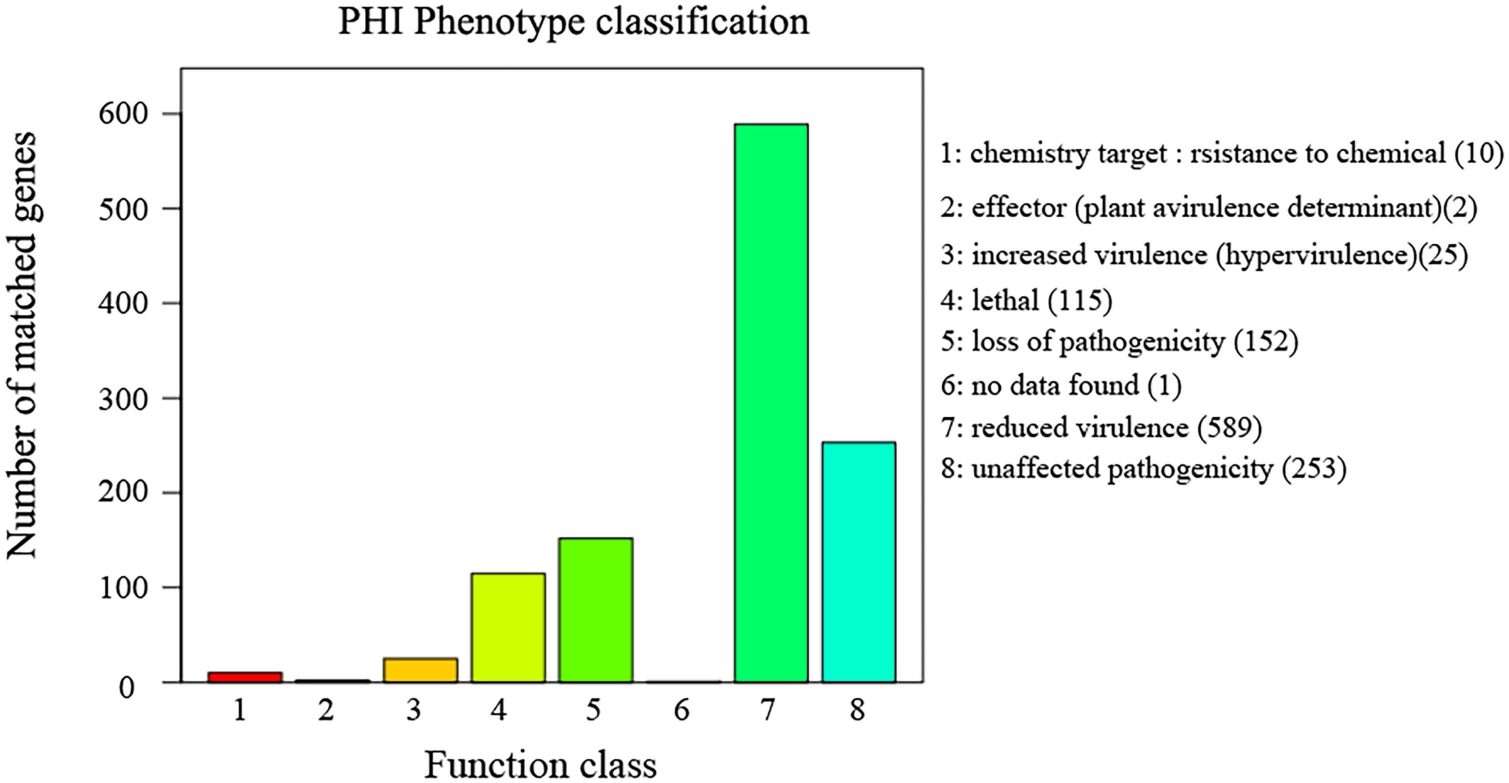
Distribution diagram of PHI phenotypic mutation type in the sample Y5 gene function annotation. PHI (Pathogen Host Interactions Database), pathogen-host interactions database, which is mainly derived from fungal, oomycete and bacterial pathogens and infects hosts including animals, plants, fungi as well as insects. This database is important for finding target genes for drug intervention studies, and it also includes antifungal compounds and their corresponding target genes. The horizontal coordinate indicates the type of phenotypic mutation and the vertical coordinate indicates the number of genes on the annotation.

The annotation result statistics of the encoded genes were shown in the Supplementary Table S2, and 9.4% (3,531 genes) could be annotated and classified into different functional categories using COG (Cluster of Orthologous Groups of proteins; Figure 4), and 3,531 predicted genes could be assigned to 23 COG families. Except for the genes predicted to have general (382 genes) or unknown functions (133 genes), the largest group of genes were involved in “translation, ribosomal structure, and biogenesis” (525 genes, 8.93%). In addition, 5,408 predicted genes had KEGG orthologs, and 4,013 predicted genes had Swiss-Prot orthologs. A total of 12,167 predicted genes had NR orthologs, the largest proportion. The PHI database (Martin et al., 2015), mainly derived from fungi, oomycetes, and bacterial pathogens, indicated that the infected hosts include animals, plants, fungi, and insects. Complete proteome of *R. oryzae* was aligned to PHI database to reveal the pathogenicity related proteins. We observed a total of 1,147 (9.05%) PHI genes were classified into different classes such as “chemistry target: resistance to chemical” “effector (plant a virulence determinant)” “increased virulence (hypervirulence)” “lethal” “loss of pathogenicity” “no data found” “reduced virulence” and “unaffected pathogenicity.” As shown in Figure 5, we observed 10 genes associated with chemistry target about resistance to chemical. Furthermore, 115 genes belonged to lethal and 25 to increased virulence class. Remaining three classes *viz.*, loss of pathogenicity, reduced virulence and unaffected pathogenicity were having 152, 589 and 253 genes, respectively. Pathogenicity related genes identified in this study have high relevance in future fungicide designing. The database is important for finding target genes for drug interventions, and it also includes antifungal compounds and corresponding target genes. The whole-genome sequence and annotation of *Rhizopus oryzae* isolate Y5 have been deposited at NCBI^3^ with accession PRJNA814049; BioSample SAMN26535981.

## Discussion

*Rhizopus oryzae* is a destructive fungal pathogen during flue-cured tobacco. The pathogen’s extensive host range includes Rosaceae, Cucurbitaceae, Solanaceae, Brassicaceae, and Umbelliferae (Xu et al., 2020). Many molecular biology, genetic, and genomic studies have been conducted on *R. oryzae* (Ellis, 1985; Ma et al., 2009). PMs have been used to analyze many microorganisms’ phenotypes, including *Botrytis cinerea* (Wang et al., 2018b), *Phytophthora parasitica* (Wang M. S. et al., 2015), and *Alternaria alternata* (Wang H. C. et al., 2015), comparisons were made with these pathogens. In this study, the metabolic ability of a *R. oryzae* isolate obtained from flue-cured tobacco was systematically studied using PMs, and important metabolic diversity information was obtained. So far, many Zygomycetes genomes have been sequenced and are publicly available, including *R. oryzae* from different hosts. We sequenced the genome of strain Y5 from flue-cured tobacco using Illumina HiSeq and Pacific Biosciences (PacBio) technologies. The genome combined with the metabolic phenotype of the pathogen could provide a reference for the study of fungal biological characteristics and population genetic diversity. Genome sequencing can reveal role of each individual gene and their networks responsible for plant pathogen interaction, growth, evolutionary relationship and genes for pathogenicity (Iquebal et al., 2017). Whole genome sequencing of *R. oryzae* is imperative not only to study the host-pathogen (HP) interaction but such knowledge discovery may lead to more effective disease combating strategy. Annotated genes/ predicted proteins can be directly used as new targets in fungicides development using computational approach (Acero et al., 2011).

Our study revealed that *R. oryzae* had a comparatively narrow utilization range of carbon sources. In comparison to carbon, nitrogen utilization efficiency was comparatively higher, which explained the *R. oryzae* infection mechanism for tobacco. Higher levels of an unutilized carbon source or less accessible nitrogen sources may affect pathogen infection and subsequently restrain disease. The PM 4 and PM 6–8 plates showed high levels of metabolic activity; this is comparable to findings from other researchers studying *R. oryzae* (Wang et al., 2018a). In our study, for the carbon or nitrogen substrates, carbohydrates or amino acids and peptides were greatly utilized by *R. oryzae*. The utilized substrates may sustain the continued existence of *R. oryzae* in different hosts and thus affect the pathogenicity of the pathogen. Compared with other pathogens, *A. alternata* had a comparatively small range of accessible carbon compounds, and most nitrogen, sulfur, and phosphorus sources are metabolized. *Pseudomonas syringae* had a comparatively small range of accessible carbon compounds, and most nitrogen, sulfur, and phosphorus sources could not be metabolized. *B. cinerea* had a small range of accessible carbon compounds with different crops, and most nitrogen, sulfur, and phosphorus sources were metabolized comparatively. Most pathogens made full use of nitrogen sources and used carbon sources to a larger or smaller extent. The prevention and control of tobacco pole rot through exploring the absorption and utilization of different nutritional elements by the pathogenic fungus, combined with the nutritional elements absorbed by tobacco, are discussed further.

We report the genomic analysis of *R. oryzae*, one of the most widely used extracellular enzyme producers, which could also cause disease in plants and humans. A genome sequence of 50.3 Mb was assembled into 41 contigs with an N50 length of 1,791,927 bp, maximum contig length of 3,223,184 bp, and total contig length of 50,257,186 bp. Genome information of all *R. oryzae* strains is supplied in Supplementary Table S1. Small differences were recorded in the genome sizes of the *R. oryzae* strains, ranging from 37.5–55.8 Mb except for strain GL39 (72.36 Mb), and the average GC content of the genomes was 34.7%, which was lower than that of strain Y5 of *R. oryzae* GC content. The genome host of the *R. oryzae* strains were “Homo sapiens,” “urine,” and “unknown.” Most of the strains were derived from the medical environment, for instance, “lung transplant,” “sinus,” “tracheal biopsy,” “nasal cavity,” “bone marrow,” “ethmoid sinus of diabetic” and “bronchial wash.” The Y5 strain isolated from tobacco was infrequently mentioned in genomic. In this study, the total number of predicted protein-coding genes was 12,680, compared to approximately 14,000 as the highest number from other strains, with a total length of 17,290,559 bp, average gene length of 1,364 bp, and gene length/genome (%) of 34.4%. Pathogenicity related genes identified in this study have high relevance in future fungicide designing using PHI database (Cools and Hammond-Kosack, 2013). Our enlisted lethal and virulence genes can be used in future research of fungal disease management, especially by designing of new generation genomic based fungicide (Seringhaus et al., 2006), the number of lethal genes in our study was found to be 115. In other ascomycotina, for example in *S. cerevisiae*, it was found to have 900 lethal genes (Miklos and Rubin, 1996).

The characteristics of each strain depend on which genes had changed and how that affected the phenotype. Although numerous studies had investigated the genome-wide properties of *R. oryzae* and the differences between multiple strains, few reports had compared the strain’s genome with its metabolic phenotype, despite advances in genetic techniques. Further studies of the effects of such metabolism in strains using gene knockout methods are needed. The deluge of data generated by genome sequencing has led to an increasing reliance on bioinformatic predictions, since the traditional experimental approach of characterizing gene function one at a time cannot possibly keep pace with the sequence-based discovery of novel genes (Johnson et al., 2008). One common application of the PM system is to detect phenotypic changes associated with gene knockouts, and this strategy has been used to study gene function and to assess and improve genome annotation (Loh et al., 2006). We have further explored the linkage between metabolic phenotypes and genomes in the next study. *Aspergillus luchuensis* (Hong et al., 2013) could grow on carbon sources other than glucose. Further RNA sequencing studies are needed to provide quantitative evidence to support our results and elucidate expression levels under stress conditions. In addition, Orthologous Groups (OGs) containing multiple genes also affect the traits of the lines; thus, these OGs should be further studied. Further Studies to know the relationship between metabolic activities under stress conditions are needed. This study demonstrated the power of whole genome analysis to reveal novel gene sequences between different strains. Whole genome analysis of *R. oryzae* should reveal the unique genes and non-coding regions of each strain. Therefore, the genome sequences of *R. oryzae* provided a unique resource for studying pathogenicity determinants in this pathogen, understanding host adaptation mechanisms, and designing specific disease management control strategies.

## Conclusion

This study demonstrates that phenotypic characterization of a strain of the pathogen *R. oryzae* isolated from tobacco was conducted to provide basic biological and pathological information using Biolog Phenotype MicroArray (PM). In addition, the Y5 strain of *R. oryzae* was sequenced using Illumina HiSeq and Pacific Biosciences (PacBio) technologies. It indicated that *R. oryzae* could metabolize 54.21% of tested carbon sources, 86.84% of nitrogen sources, 100% of sulfur sources, and 98.31% of phosphorus sources. And also, genome sequencing results showed that the *R. oryzae* Y5 strain had raw data assembled into of 2,271 Mbp with an N50 value of 10,563 bp. A genome sequence of 50.3 Mb was polished and assembled into 53 contigs with an N50 length of 1,785,794 bp, maximum contig length of 3,223,184 bp, and a sum of contig lengths of 51,182,778 bp. A total of 12,680 protein-coding genes were predicted. This study demonstrated the power of whole genome analysis to reveal novel gene sequences between different strains. Whole genome analysis of *R. oryzae* should reveal the unique genes and non-coding regions of each strain. Therefore, the genome sequences of *R. oryzae* provided a unique resource for studying pathogenicity determinants in this pathogen, understanding host adaptation mechanisms, and designing specific disease management control strategies.

## Data availability statement

The datasets presented in this study can be found in online repositories. The names of the repository/repositories and accession number(s) can be found at: https://www.ncbi.nlm.nih.gov/, PRJNA814049.

## Author contributions

ZL, C-hS, YH, H-cW, W-hL and L-tC contributed to conceptualize and design of the study. ZL organized the database and performed the statistical analysis. ZL and H-cW wrote the first draft of the manuscript. H-cW and YH revised the manuscript and wrote some sections. All authors contributed to the article and approved the submitted version.

## Funding

This work was supported by the China National Tobacco Corporation [110202101048(LS-08), 110202001035(LS-04)], the National Natural Science Foundation of China (31960550, 32160522), the Hundred Level Innovative Talent Foundation of Guizhou Province [GCC(2022)028–1], the Guizhou Science Technology Foundation (ZK[2021]Key036), the International Science and Technology Cooperation Base ([2020]4102), Guizhou Provincial Academician Workstation of Microbiology and Health([2020]4004), and the Guizhou Tobacco Company (2020XM22, 2020XM03). The authors declare that this study received funding from the China National Tobacco Corporation and Guizhou Tobacco Company. The funders were not involved in the study design, collection, analysis, interpretation of data, the writing of this article or the decision to submit it for publication. All authors declare no other competing interests.

## Conflict of interest

The authors declare that the research was conducted in the absence of any commercial or financial relationships that could be construed as a potential conflict of interest.

## Publisher’s note

All claims expressed in this article are solely those of the authors and do not necessarily represent those of their affiliated organizations, or those of the publisher, the editors and the reviewers. Any product that may be evaluated in this article, or claim that may be made by its manufacturer, is not guaranteed or endorsed by the publisher.

## Supplementary material

The Supplementary material for this article can be found online at: https://www.frontiersin.org/articles/10.3389/fmicb.2022.1031023/full#supplementary-material

SUPPLEMENTARY FIGURE S1 Evolutionary relationships of *Rhizopus oryzae.* Phylogenetic tree showing the phylogenetic relationship amongst difference belonging to the species *Rhizopus oryzae* (*Rhizopus oryzae*, also known as *Rhizopus arrhizus*), is a filamentous fungus that is the most common cause of mucormycosis, also referred to as zygomycosis. An opportunistic pathogen, *R. oryzae* causes disease primarily in immunocompromised people, such as those with diabetes mellitus, cancer, or AIDS. *R. oryzae* is found in soil, decaying fruit and vegetables, old bread, and animal dung. It is used in the preparation of fermented foods and alcoholic beverages in Asia. *R. oryzae* is also a destructive pathogen that frequently causes tobacco pole rot in curing chambers.

## Footnotes

^1^ http://www.repeatmasker.org/

^2^ https://www.pacb.com/support/software-downloads/

^3^ https://www.ncbi.nlm.nih.gov/
