# Supplementary figures and images for: Phenotypic analysis and genome sequence of Rhizopus oryzae strain Y5, the causal agent of tobacco pole rot
Source: Front Microbiol. 2023 Jan 4;13:1031023. doi: 10.3389/fmicb.2022.1031023 (PMC9846616; doi:10.3389/fmicb.2022.1031023)

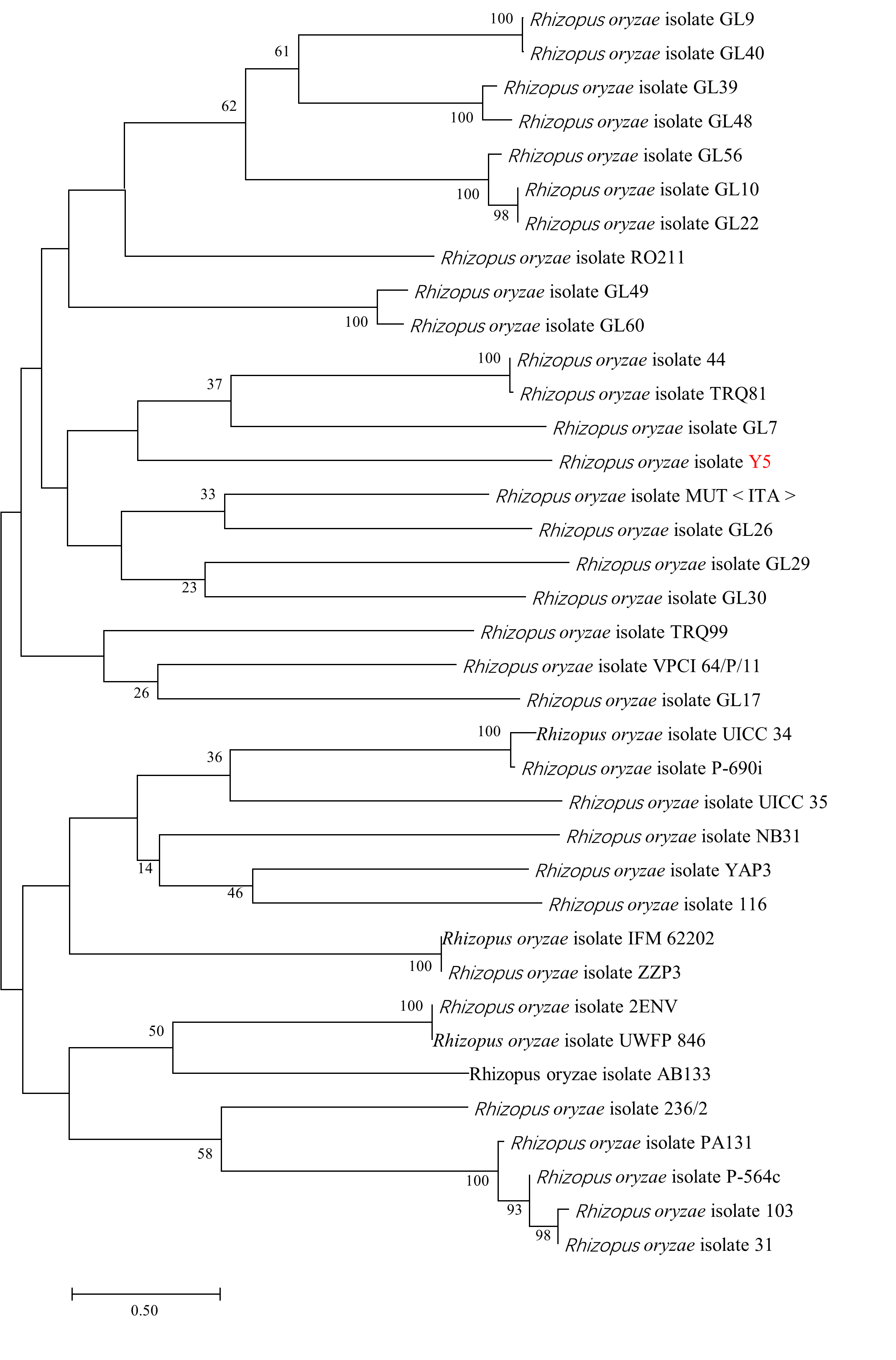

Supplement: SUPPLEMENTARY FIGURE S1 — Evolutionary relationships of Rhizopus oryzae. Phylogenetic tree showing the phylogenetic relationship amongst difference belonging to the species Rhizopus oryzae (Rhizopus oryzae, also known as Rhizopus arrhizus), is a filamentous fungus that is the most common cause of mucormycosis, also referred to as zygomycosis. An opportunistic pathogen, R. oryzae causes disease primarily in immunocompromised people, such as those with diabetes mellitus, cancer, or AIDS. R. oryzae is found in soil, decaying fruit and vegetables, old bread, and animal dung. It is used in the preparation of fermented foods and alcoholic beverages in Asia. R. oryzae is also a destructive pathogen that frequently causes tobacco pole rot in curing chambers. [file Image_1.TIF]
