# Supplementary material for: Phenotypic analysis and genome sequence of Rhizopus oryzae strain Y5, the causal agent of tobacco pole rot
Source: Front Microbiol. 2023 Jan 4;13:1031023. doi: 10.3389/fmicb.2022.1031023 (PMC9846616; doi:10.3389/fmicb.2022.1031023)
Supplement: Supplementary file 2 [file Table_1.docx]

**Supplementary table 1.** Summary of several main features for *R. oryzae* strain Y5 and forty-three sequenced *Rhizo­pus oryzae* genomic data

| **Species** | **Strain** | **BioProject** | **Level** | **Size(Mb)** | **GC%** | **CDS** | **No. gene (coding)** | **Host** | **Isolation source** |
| --- | --- | --- | --- | --- | --- | --- | --- | --- | --- |
| *R. oryzae* | Y5 | PRJNA814049 | Contig | 50.3 | 35.6 | 37383 | 12,680 | tobacco | Environment |
| *R. oryzae* | R2701 | PRJNA833221 | Scaffold | 40 | 34.6 | Unkown | Unkown | Homo sapiens | Unkown |
| *R. oryzae* | 97-1192 | PRJNA186024 | Contig | 42.9 | 35.3 | Unkown | Unkown | Homo sapiens | bronchial wash |
| *R. oryzae* | PG1902 | PRJNA833221 | Scaffold | 36.8 | 35.2 | Unkown | Unkown | Homo sapiens | Unkown |
| *R. oryzae* | GL49 | PRJNA475137 | Scaffold | 42.2 | 35 | 14415 | 14421 | NA | Environment |
| *R. oryzae* | GL56 | PRJNA475137 | Scaffold | 42.2 | 35 | 14291 | 14298 | Homo sapiens | Unkown |
| *R. oryzae* | GL38 | PRJNA475137 | Scaffold | 42.2 | 35 | 14318 | 14326 | NA | Environment |
| *R. oryzae* | GL32 | PRJNA475137 | Scaffold | 42.2 | 35 | 14407 | 14415 | NA | air |
| *R. oryzae* | GL34 | PRJNA475137 | Scaffold | 42.1 | 35 | 14265 | 14273 | Homo sapiens | Sinus |
| *R. oryzae* | GL45 | PRJNA475137 | Scaffold | 42.4 | 35 | 14369 | 14376 | NA | Environment |
| *R. oryzae* | GL40 | PRJNA475137 | Scaffold | 43.2 | 34.8 | 14375 | 14383 | NA | Environment |
| *R. oryzae* | GL41 | PRJNA475137 | Scaffold | 42.1 | 35 | 14439 | 14445 | NA | Environment |
| *R. oryzae* | GL53 | PRJNA475137 | Scaffold | 42.2 | 35 | 14383 | 14389 | NA | Environment |
| *R. oryzae* | GL44 | PRJNA475137 | Scaffold | 42.1 | 35 | 14338 | 14344 | NA | air |
| *R. oryzae* | GL37 | PRJNA475137 | Scaffold | 42.2 | 35 | 14392 | 14397 | NA | Environment |
| *R. oryzae* | GL52 | PRJNA475137 | Scaffold | 42.1 | 35 | 14330 | 14336 | NA | Environment |
| *R. oryzae* | GL46 | PRJNA475137 | Scaffold | 42 | 35 | 14247 | 14254 | NA | Environment |
| *R. oryzae* | GL31 | PRJNA475137 | Scaffold | 42.3 | 35 | 14441 | 14448 | NA | air |
| *R. oryzae* | GL60 | PRJNA475137 | Scaffold | 42.2 | 34.8 | 14154 | 14162 | Homo sapiens | Homo sapiens |
| *R. oryzae* | GL43 | PRJNA475137 | Scaffold | 42.3 | 35 | 14344 | 14351 | NA | Environment |
| *R. oryzae* | GL42 | PRJNA475137 | Scaffold | 43.8 | 34.4 | 14329 | 14336 | NA | Environment |
| *R. oryzae* | GL36 | PRJNA475137 | Scaffold | 42.2 | 34.8 | 14269 | 14278 | Homo sapiens | Homo sapiens |
| *R. oryzae* | GL30 | PRJNA475137 | Scaffold | 45.1 | 36.9 | 15802 | 15808 | NA | Environment |
| *R. oryzae* | GL29 | PRJNA475137 | Scaffold | 43.4 | 35.8 | 14774 | 14778 | NA | Environment |
| *R. oryzae* | GL20 | PRJNA475137 | Scaffold | 42.6 | 35.9 | 14331 | 14337 | NA | Environment |
| *R. oryzae* | GL26 | PRJNA475137 | Scaffold | 44 | 36.7 | 15264 | 15271 | NA | Environment |
| *R. oryzae* | GL17 | PRJNA475137 | Scaffold | 46.1 | 37.8 | 17009 | 17015 | NA | Environment |
| *R. oryzae* | GL16 | PRJNA475137 | Scaffold | 47.2 | 35.7 | 14811 | 14816 | NA | Environment |
| *R. oryzae* | GL3 | PRJNA475137 | Scaffold | 48 | 38.4 | 18266 | 18273 | NA | Environment |
| *R. oryzae* | GL10 | PRJNA475137 | Scaffold | 45.2 | 36.8 | 15746 | 15753 | NA | Environment |
| *R. oryzae* | GL6 | PRJNA475137 | Scaffold | 47.3 | 38 | 17556 | 17561 | NA | Environment |
| *R. oryzae* | GL22 | PRJNA475137 | Scaffold | 48.2 | 39 | 18996 | 19001 | NA | Environment |
| *R. oryzae* | GL2 | PRJNA475137 | Scaffold | 49 | 38.7 | 19020 | 19026 | NA | Environment |
| *R. oryzae* | GL7 | PRJNA475137 | Scaffold | 50.7 | 40.4 | 21978 | 21982 | NA | Environment |
| *R. oryzae* | GL48 | PRJNA475137 | Scaffold | 55 | 34.7 | 16170 | 16174 | NA | Environment |
| *R. oryzae* | GL9 | PRJNA475137 | Scaffold | 44.9 | 38 | 16121 | 16124 | NA | Environment |
| *R. oryzae* | Type I NRRL 21396 | PRJNA186015 | Contig | 42.8 | 35.2 | Unkown | Unkown | Homo sapiens | ethmois sinus of diabetic |
| *R. oryzae* | 99-133 | PRJNA186016 | Contig | 41.5 | 35.4 | Unkown | Unkown | Homo sapiens | bone marrow |
| *R. oryzae* | B7407 | PRJNA184879 | Contig | 43.3 | 34.9 | Unkown | Unkown | Homo sapiens | nasal cavity |
| *R. oryzae* | Type I NRRL 13440 | PRJNA186013 | Contig | 43.4 | 35.2 | Unkown | Unkown | Homo sapiens | tracheal biopsy |
| *R. oryzae* | Type I NRRL 18148 | PRJNA186014 | Contig | 47.5 | 35 | Unkown | Unkown | Homo sapiens | sinus |
| *R. oryzae* | HUMC 02 | PRJNA186018 | Scaffold | 40.3 | 34.7 | Unkown | Unkown | Homo sapiens | sinus |
| *R. oryzae* | 99-892 | PRJNA186020 | Scaffold | 39.1 | 35.4 | Unkown | Unkown | Homo sapiens | lung transplant |
| *R. oryzae* | GL39 | PRJNA475137 | Scaffold | 72.4 | 36.9 | 29149 | 29158 | NA | Environment |

**Supplementary table 2.** Statistics of annotation results of coding genes performed by several general function databases

| Sample ID | Y5^*^ |
| --- | --- |
| {nr} | 12,167 |
| {SwissProt} | 4,013 |
| {KEGG} | 5,408 |
| {KOG} | 3,531 |
| {TCDB} | 514 |
| {GO} | 9,167 |
| {PHI} | 1,147 |
| {VFDB} | 95 |
| {DFVF} | 578 |
| {ARDB} | 0 |
| {P450} | 55 |
| {Secretory_Protein} | 452 |
| {CAZy} | 256 |

^*^The columns in the table indicate the number of genes encoding genes annotated to each database.
